# Effect of nanocomposite alginate‐based film incorporated with cumin essential oil and TiO_2_ nanoparticles on chemical, microbial, and sensory properties of fresh meat/beef

**DOI:** 10.1002/fsn3.2724

**Published:** 2022-04-05

**Authors:** Mehran Sayadi, Ali Mojaddar Langroodi, Sedigheh Amiri, Mohsen Radi

**Affiliations:** ^1^ Department of Food Safety and Hygiene School of Health Fasa University of Medical Sciences Fasa Iran; ^2^ Department of Food Science and Technology Yasooj Branch Islamic Azad University Yasooj Iran; ^3^ Sustainable Agriculture and Food Security Research Group Yasooj Branch Islamic Azad University Yasooj Iran

**Keywords:** active packaging, cumin essential oil, meat quality, nanocomposite alginate‐based film, TiO_2_ nanoparticles

## Abstract

The environmental problems of synthetic plastics in food packaging have led researchers to synthesize biodegradable films. In this study, nanocomposite alginate‐based films containing TiO_2_ nanoparticles (1%) and cumin essential oil (CEO, 2%) were fabricated and the potential of these films to protect beef from chemical [pH, total volatile base nitrogen (TVBN), peroxide value, and thiobarbituric acid reactive substances (TBA)] and microbial [total viable count, *Enterobacteriaceae*, lactic acid bacteria, *Listeria monocytogenes*, and *Pseudomonas* spp.] spoilage was evaluated during 24 days of storage (4°C). The active films significantly induced the reduction in lipid oxidation, microbial growth, and TVBN values, improved the sensory attributes of treated samples, maintained the redness of meats for a longer time, and increased the shelf life of beef from 4 to 16 days. The results of this study showed that TiO_2_/CEO alginate‐based nanocomposite film has a great potential for application in meat and meat products.

## Graphical abstract

Cumin essential oil and TiO_2_ nanoparticles were incorporated into the alginate‐based film. The active packaging reduced the beef microbial growth and fat oxidation remarkably. The functional ingredients could quadruple the shelf life of fresh beef.

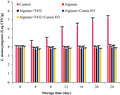

## 1 INTRODUCTION

In general, meat‐based foods have very low microbial and oxidative stability and are easily exposed to microbial and chemical spoilage during the production and storage chain (Mojaddar Langroodi et al., 2021). Therefore, improving the storage life of fresh meat is one of the important challenges of the meat industry which has attracted the attention of many researchers in recent years (Junior et al., 2020; Sepahvand et al., 2021). In this regard, the use of biopolymeric‐based coatings and films is proposed as an important solution due to their barrier, mechanical, optical, and biodegradability characteristics (Bagheri et al., 2019a; Molayi et al., 2018). Sodium alginate is a biopolymer with a high potential for being used as the basic component of films and coatings due to its unique properties such as biodegradability, low cost, availability, ease of use, nontoxicity, stability, and gel formation in the presence of polyvalent cations (Bagheri et al., 2019b; Karimi Khorrami et al., 2021; Nehchiri et al., 2021). The use of biopolymers in the forms of coating and film can restrict oxygen availability and prevent moisture loss, and thereby can increase the shelf life of many products in many researches (Bagheri et al., 2020; Martiny et al., 2020; Radi, Akhavan‐Darabi, et al., 2017; Radi, Firouzi, et al., 2017). The effectiveness of such coatings can be remarkably increased by incorporating antimicrobial and antioxidant compounds in their matrix to maintain high concentrations of these substances on the surface of coated products which are more susceptible to bacterial infestation (Akhavan et al., 2021). Different compounds like green and black tea extracts (Amiri et al., 2018; Radi, Firouzi, et al., 2017), *Thymus vulgaris* EO (Almasi, Radi, Amiri, 2020), polylactic acid (Guo et al., 2014), orange peel EO microemulsion (Radi, Akhavan‐Darabi, et al., 2017), (hot) acetic acid (Hosseini‐Farahi et al., 2018; Radi et al., 2010), calcium sulfate (Hosseini‐Farahi et al., 2016), and salycilic acid (Amiri et al., 2021; Hosseinifarahi et al., 2020), and treatments like UV irradiation (Abdipour et al., 2020) have been incorporated into the biopolymeric‐active packagings to enhance the antimicrobial activity of the films. In this regard, the use of essential oils (EOs) has received much attention due to their naturality and high antimicrobial activity (Najjaa et al., 2020; Radi, Akhavan‐Darabi, et al., 2017). Cumin is known for its antimicrobial, nutritional value, antioxidant, and pharmaceutical (antihypertensive, anticancer, etc.) properties, which are mainly associated with its EO (CEO) (Haghiroalsadat et al., 2010). The main components of CEO are cuminol, cumin aldehyde, menthon derivatives, and γ‐terpinene, which are responsible for its odor and biological effects. The antimicrobial effects of CEO on foodborne pathogens in different food products have been well demonstrated (Hyldgaard et al., 2012; Sharafati Chaleshtori et al., 2016; Taheri et al., 2018).

Titanium dioxide (TiO_2_), also known as titanium oxide, has many applications in today's modern world, and is mainly used as a pigment in different industries (Hur et al., 2005). The US Food and Drug Administration has approved the use of TiO_2_ as a harmless color additive in food, medicine, and cosmetic products (Alizadeh‐Sani et al., 2018). Extensive researches have been performed on the antimicrobial effect of TiO_2_ on a wide range of living organs including viruses, bacteria, fungi, algae, and cancer cells (Paspaltsis et al., 2006). The photocatalytic reaction of TiO_2_ is one of its known natural effects in reducing fungal contamination. Improving the application properties of biopolymer films by metal nanoparticles of TiO_2_ has been considered by researchers in recent years (Alizadeh‐Sani et al., 2018; Azizi‐Lalabadi et al., 2020; Marcous et al., 2017). In this study, the potential application of alginate‐based film containing TiO_2_ nanoparticles and CEO as functional ingredients on chemical, microbial, and physical properties of beef was evaluated during cold storage.

## 2 MATERIALS AND METHODS

### 2.1 Materials

TiO_2_ nanoparticles (anatase) with purity of more than 99% and particle size of 10–25 nm were obtained from US Research Nanomaterials, Inc. Na‐alginate was obtained from Behin Azma Co. Nutrient broth, Violet Red Bile Agar (VRBA), Cetrimide fucidin cephaloridine agar (CFC agar), De Man, Rogosa, Sharpe agar (MRS), Listeria Chrom agar, and plate count agar (PCA) were purchased from Merck Chemical Co.

### 2.2 The extraction of CEO

The CEO was extracted by the steam distillation method with the seed/water ratio of 1:5 (w/v) for 4 h by using a Clevenger apparatus. The yield and density of obtained CEO were 0.5% (v/w) and 0.878 (at 20°C), respectively (Sharafati Chaleshtori et al., 2016).

### 2.3 Chemical composition of CEO

A capillary gas chromatography (GC–MS; Hewlett Packard 6890) and a mass spectroscopy (Hewlett Packard 6890) were used to analyze the chemical composition of CEO. A capillary column with 0.32 mm diameter and length of 30 mm was used for the analysis. The analysis conditions were defined as follows: oven temperature (60°C), the column temperature (60°C—for the first 3 min after the injection, which was raised to 220°C with a rate of 6°C/min), the injection temperature (250°C), the flow rate of helium as the carrier gas (1 ml/min), and the MS ionization voltage (70 electronvolt; Amiri et al., 2013).

### 2.4 Alginate film preparation

To prepare the alginate film, 4 g of Na‐alginate was dissolved in 200 ml distilled water and stirred (200 rpm, for half an hour) at 60°C [to prevent lump formation, Na‐alginate powder was added gradually to the stirring water, and time was given for the Na‐alginate to be dissolved during the addition of powder (Radi & Amiri, 2013)]. Thereafter, 1 g glycerol was added to the film solution as a plasticizer. One percent of TiO_2_ nanoparticles (Al + TiO_2_), 2% CEO (Al + CEO), and a mixture of 1% TiO_2_ and 2% CEO (Al + TiO_2_ + CEO) were added, separately, as antimicrobial and antioxidant agents to prepare different treatments. Then, an ULTRA‐TURRAX^®^ homogenizer (T18; IKA) was used to homogenize the solution (6000 rpm, 10 min). The prepared solutions were deaerated (at 40°C, 5 min) by a rotary vacuum evaporator (RV‐10 control; IKA). Then, 20 g of the solutions was poured into polystyrene Petri dishes (11 cm diameter) and dried in an incubator at 25°C with 40% relative humidity for 24 h (Nehchiri et al., 2021).

### 2.5 Samples preparation

After purchasing the beef from a local slaughterhouse, the meat was transferred to the laboratory under aseptic conditions (pH = 5.70 ± 0.04), cut into pieces with a thickness of 2 cm and a length of 20 cm, and then wrapped with the preprepared films. The treatments were defined as follows: Al, Al + TiO_2_, Al + CEO, and Al + TiO_2_ + CEO, as well as a control sample which was not wrapped within an Al‐based film. The wrapped meat samples were transferred to the polyethylene plastic bags and stored in a refrigerator (4 ± 1°C) for 24 days (Almasi et al., 2021). The sampling was performed at 4‐day intervals for microbiological, physical, chemical, and sensory experiments. Five Al‐based wraps containing meat samples were placed in a tray to be used as a replicate for each measurement time. Regarding the presence of seven measurement times (throughout the storage time), 35 plastic wraps were considered for the total measurement time of 24 days for one replicate and a total of three replicates were considered for each treatment.

### 2.6 Microbial analyses

To count the microbial population, 10 g of each sample was aseptically weighed, diluted, and homogenized (Seward Stomacher 400) with 50 ml of sterile saline solution (0.9%). After serial dilution, 0.1 ml of the desired dilutions prepared from the relevant treatments was cultured aerobically on selective media for determination of lactic acid bacteria (LAB, MRS), total mesophilic bacterial count (TVC, PCA), *Enterobacteriaceae* (VRBA), *Pseudomonas* spp. (CFC agar), and *L. monocytogenes* (Listeria Chrom agar) and incubated for 24 h at 37°C for PCA, VRBA, and Listeria Chrom agar, 24–72 h at 37°C for MRS agar, and 48 h at 20°C for CFC (CLSI, 1999).

### 2.7 The measurement of pH

Ten grams of meat samples was added to 90 ml of deionized water and homogenized (1000 rpm) for 1 min. After 10 min, the pH of the homogenate was measured using a pH meter (CG824, Schott pH meter; AOAC, 2000).

### 2.8 Chemical properties

The macrodistillation method was used to measure the total volatile base nitrogen (TVBN) content of the meat samples (Kirk & Sawyer, 1991). A colorimetric method was used for the measurement of thiobarbituric acid (TBA; Barbin, 1975). The measurement was performed at 538 nm using a spectrophotometer (T80+, PG Instruments). The results are reported as mg malonaldehyde (MDA)/kg sample. To measure the peroxide value (PV) of the beef samples, the iodometric method based on the titration of the samples with thiosulfate solution was performed (AOAC, 2000).

### 2.9 Color measurement

A digital colorimeter (CR‐400, Konica Minolta Sensing Inc.) was used to measure the color of meat samples. For this purpose, the colorimeter was first calibrated by a standard white plate (L* = 95.44, a* = −0.47, b* = 2.51). Then, the meat samples were placed on the standard white plate and the color coordinates values [L* (lightness), a* (green–red), and b* (blue–yellow)] were measured (Bagheri et al., 2014; Radi et al., 2010).

### 2.10 Sensory evaluation

The sensory evaluation was conducted according to the method described by Sharafati Chaleshtori et al. (2016) with some modifications. The taste panels consisted of 30 semi‐trained panelists (15 males and 15 females; aged 20–45 years; mean age, 36 years). Each panelist was asked to assess all of the samples at the same session. The meat odor, color, and overall acceptance were assessed using a 9‐point hedonic scale (dislike extremely [1], dislike very much [2], dislike moderately [3], dislike slightly [4], neither like nor dislike [5], like slightly [6], like moderately [7], like very much [8], and like extremely [9]). The whole process was performed at ambient temperature and standard lighting.

### 2.11 Statistical analysis

Statistical analyses were conducted by running one‐way analysis of variance (ANOVA), using MSTAT‐C. The comparison of means was performed by Duncan's multiple‐test range at *p* < .05.

## 3 RESULT AND DISCUSSIONS

### 3.1 Chemical composition of CEO

The main components of CEO, as it is shown in Table 1, are cuminal (43.4%), γ‐terpinene (32.62%), 1‐methyl‐2‐(1‐methylethyl)benzene (9.32%), pinocarveol (4.21%), copaene (3.86%), linalool (2.11%), and 1‐methyl‐4‐(1‐methylethyl)‐1,4‐cyclohexadiene (1.02%). The dominant compounds of CEO are the oxygenated monoterpenes, followed by the hydrocarbon monoterpenes (Abbdellaoui et al., 2019). The antimicrobial activity of CEO is largely attributed to its cuminal component (Wannera et al., 2010). The results of this study are in good agreement with the results of El‐Ghorab et al. (2010), who confirmed that cuminal (27.7%), γ‐terpinene (23.7%), pinocarveol (11.4%), and 1‐methyl‐2‐(1‐methylethyl)benzene (7.7%) are the major components of CEO. However, Abbdellaoui et al. (2019) demonstrated that cumin aldehyde (30%–33%), γ‐terpinen‐7‐al (20%–28%), α‐terpinen‐7‐al (13%), and γ‐terpinene (6%–12%) are the main components of CEO. The differences in components and quantities of different reports might refer to the different cultivation and climatic conditions as well as the environment which affects the chemical composition of the EO (El‐Ghorab et al., 2010).

**TABLE 1:** Major chemical compositions of cumin essential oil

Compound	RT (Min)^a^	Percent^b^
Cuminal	17.238	43.4
γ‐Terpinene	22.325	32.62
1‐Methyl‐2‐(1‐methylethyl)benzene	11.271	9.32
Pinocarveol	12.529	4.21
Copaene	13.422	3.86
Linalool	23.138	2.11
1‐Methyl‐4‐(1‐Methylethyl)‐1,4‐Cyclohexadiene	14.261	1.02
Sabinene	17.239	0.72
β‐Terpineol	21.251	0.31
Carotol	12.427	0.24
(5R)‐5‐Methyl‐2‐(1‐methylethylidene)cyclohexanone	17.362	0.16
α‐Pinene	13.218	0.12
6,6‐Dimethyl‐2‐methylene‐bicyclo[2.2.1]heptan‐3‐one	22.458	0.5
Total identified		98.59

^a^ Retention time.

^b^ Relative proportions as percent of the total peak area.

### 3.2 Microbial analyses

**FIGURE 1:**
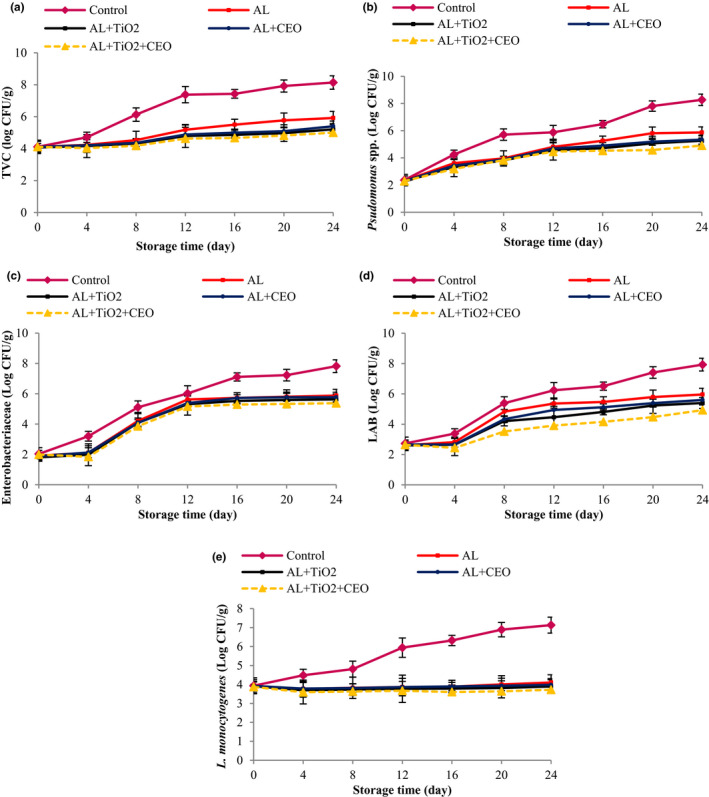
Total viable count (a), *Pseudomonas* spp. (b), *Enterobacteriaceae* (c), lactic acid bacteria (d), and *Listeria monocytogenes* (e) of beef packed in alginate‐based films containing TiO_2_ and CEO at 4°C during 24 days of storage

The meat samples wrapped with Al films were evaluated for total mesophilic bacteria, *Enterobacteriaceae*, LAB, *pseudomonas* spp., and *L. monocytogenes*. The results are shown in Figure 1. According to the results, in all samples, an increasing trend was observed for bacterial growth over time. Meanwhile, the control sample had the highest bacterial growth and the population of all studied bacteria in this sample increased from almost 2.37 to about 8.00 log CFU/g after 24 days of storage. The usage of all Al films slowed down the bacterial growth trend significantly throughout the storage time. In this regard, Al + TiO_2_ + CEO sample showed the greatest decrease in the microbial growth, followed by Al + TiO_2_ and Al + CEO samples, respectively (*p* <0.05). The lowest antimicrobial effect was related to the plain Al films. The Al + TiO_2_ + CEO films reduced the population of total mesophilic bacteria, *Enterobacteriaceae*, LAB, *Pseudomonas* spp., and *L. monocytogenes* by about 3.10, 2.44, 3.00, 3.36, and 3.41 logarithmic cycles in comparison with the control, respectively. The samples treated with Al + TiO_2_ reduced by about 2.93, 2.19, 2.53, 3.01, and 3.24 logarithmic cycles and the samples treated with Al + CEO reduced by 2.74, 2.05, 2.33, 2.93, and 3.15 logarithmic cycles the population of total mesophilic bacteria, *Enterobacteriaceae*, LAB, *pseudomonas* spp., and *L. monocytogenes* after 24 days of storage, respectively (*p* < .05). However, the samples covered with Al films reduced the population of total mesophilic bacteria, *Enterobacteriaceae*, LAB, *pseudomonas* spp., and *L. monocytogenes* by about 2.22, 1.94, 1.97, 2.40, and 3.03 logarithmic cycles compared to the control sample after 24 days of storage, respectively (*p* < .05).

For *L. monocytogenes*, the use of plain Al or (nano)composite Al films effectively reduced the population of *L. monocytogenes* from 3.93 to 3.77 (in the Al and Al + CEO samples), 3.69 (in the Al + TiO_2_ sample), and 3.64 (in the GE + TiO_2_ + CEO sample) log CFU/g until the 4th day of storage, indicating that the plain and (nano)composite Al films were initially able to reduce the *L. monocytogenes* population (*p* < .05). After that, the *L. monocytogenes* population started to increase to 4.1, 3.98, 3.89, and 3.72 log CFU/g in the treated beef samples with Al, Al + CEO, Al + TiO_2_, and Al + TiO_2_ + CEO, respectively, while the increase in *L. monocytogenes* population was from 3.93 to 7.13 log CFU/g in the control sample (*p* < .05). Results showed that *L. monocytogenes* was the most sensitive bacteria against the used treatments, as the used films were able to inhibit the growth of *L. monocytogenes* remarkably.

According to the results, all treated samples, especially those treated with CEO, TiO_2_, and TiO_2_ + CEO, could effectively reduce the population of examined G+ and G− bacteria. The Al film can act as a semipermeable film and restrict the oxygen availability on the surface of the meat samples, decreasing the microbial growth trend in comparison with the control.

The antimicrobial activity of the CEO resulted from its phenolic compounds (Alizadeh Behbahani et al., 2019). The results of GC‐MS confirmed that CEO is a monoterpenoid EO and the monoterpenes in CEO can destroy the cellular respiratory mechanisms of bacteria and ruin the transmission system of ions, resulting in cellular death. Besides, it has been reported that the phenolic compounds of CEO inhibit the growth of microorganisms through the enzymatic inhibition of the oxidized compound or reacting with sulfhydryl groups of proteins via nonspecific modes, and modifying protein functionality (Alizadeh Behbahani et al., 2019). The antifungal and antimicrobial activities of CEO have been demonstrated in several studies (Alizadeh Behbahani et al., 2019; Sharafati Chaleshtori et al., 2016).

The antimicrobial of TiO_2_ is related to the ability of this compound to generate reactive oxygen species (ROS), which disrupt the bacteria membrane or react with the cytoplasmic membranes within the bacteria cell, as well as react with the key cellular enzymes (Alizadeh‐Sani et al., 2020). Othman et al. (2014) confirmed the antimicrobial activity of TiO_2_ nanoparticles against *Escherichia coli* in the LDPE films. Feng et al. (2019) found that the whey protein nanofibrils (WPNFs)/TiO_2_ nanoparticles films limit *L*. *monocytogenes*, *Staphylococcus aureus*, *Salmonella Enteritidis*, and *E. coli* growth. It has been reported that *T. vulgaris*‐loaded microemulsions in an alginate‐based film maintained the microbial quality of ground beef (Almasi et al., 2021; Almasi, Radi, Amiri, Torri, 2020). Noshad et al. (2021) reported that application of *Plantago major* seed mucilage containing *Citrus limon* essential oil reduced the microbial growth (total viable count, psychrotrophic bacteria, *E*. *coli*, *S*. *aureus*, and fungi) significantly in buffalo meat.

As it was demonstrated, TiO_2_ antimicrobial activity was greater than that of CEO and the presence of CEO and TiO_2_ together within the nanocomposite film had a synergistic effect as the greatest reduction in the bacteria counts was observed due to the presence of the phenolic compounds of CEO as well as the production of ROS as a result of TiO_2_ nanoparticles (Alizadeh‐Sani et al., 2020). Sani et al. (2017) reported a strong antimicrobial activity against *L. monocytogenes* and *S. aureus* by rosemary oil and TiO_2_. The results of this study were confirmed by previous studies (Ghaderi‐Ghahfarokhi et al., 2016; Raeisi et al., 2016).

### 3.3 pH

**FIGURE 2:**
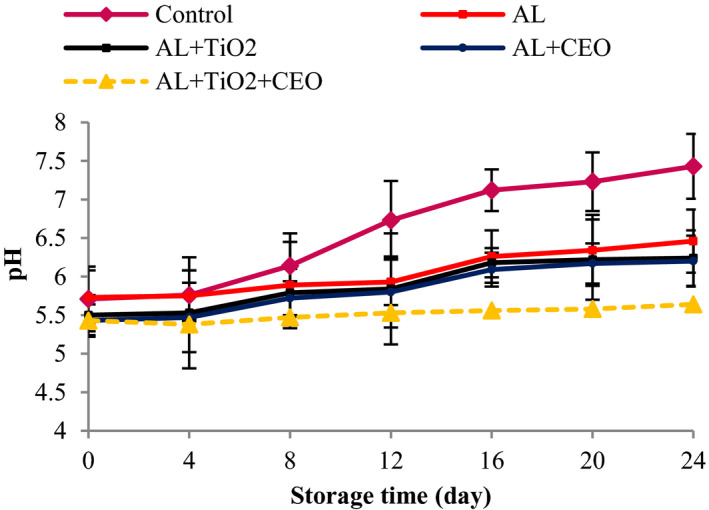
The pH changes in beef packed in alginate‐based films containing TiO_2_ and CEO at 4°C during 24 days of storage

The initial pH of beef samples was 5.70 which increased significantly to 7.43 in the control, 6.36 in Al‐coated sample, 6.22 in Al–TiO_2_‐ and Al–CEO‐treated samples, and 5.64 in Al–TiO_2_–CEO sample after 24 days of storage (*p* < .05, Figure 2). According to the results, the pH increase rate in the control sample was considerably higher than in the treated samples. The higher pH in the control sample may be attributed to a higher bacterial growth in these samples, which was demonstrated in the microbial section results. As the bacterial population increases, the amount of bacterial enzymes increases in the meat tissue. The produced enzymes begin to decompose the meat proteins and produce nitrogenous compounds, resulting in an increase in the meat pH (Sayadi, Amiri, et al., 2021). The delay in the pH increase in nanocomposite films containing TiO_2_ and CEO might be attributed to the ability of the used antimicrobial and antioxidant agents to decrease microbial growth and to inhibit the decomposition of proteins and other nitrogenous compounds, such as ammonia and trimethylamine (Alizadeh‐Sani et al., 2020). The pH value of Al–TiO_2_–CEO did not exceed the upper limit value for pH (5.80) even on day 24.

In line with our findings, Alizadeh‐Sani et al. (2020) reported an increase in pH from 5.70 to 7.45 for the control and 5.70 to 6.18 for the lamb meats packed in whey protein/cellulose nanofiber matrix films containing rosemary oil–TiO_2_. In another study, Khezrian and Shahbazi (2018) reported an increase in pH values from 5.80 to 6.60 in the camel meat treated with nanocomposite films of chitosan and carboxymethyl cellulose (CMC). The higher pH values in the control sample were observed in other studies on chicken breast meat packaged by PET/chitosan/alginate films incorporating black cumin oil (Takma & Korel, 2019) and turkey breast meat coated with chitosan containing CEO (Taheri et al., 2018).

### 3.4 Total volatile base nitrogen

**FIGURE 3:**
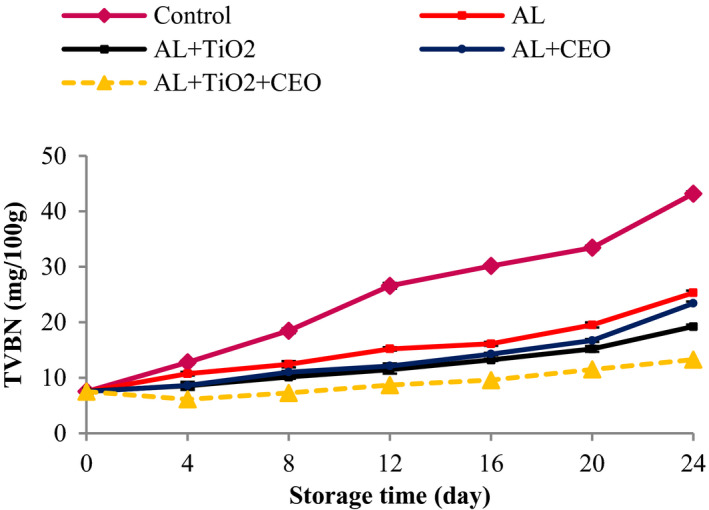
Changes in total volatile base nitrogen values of beef packed in alginate‐based films containing TiO_2_ and CEO at 4°C during 24 days of storage

The activity of the endogenous enzymes of the meat as well as the bacteria enzymes produces nitrogenous compounds which are measured in the TVBN test. Therefore, the higher values of TVBN indicate the higher activity of endogenous enzymes as well as bacterial activity that in turn is an indication of the meat spoilage (Alizadeh‐Sani et al., 2020). On day 0, the TVBN value of the meat samples was 7.51 mg N/100 g meat, indicating the good hygienic quality of meat. The TVBN values of all samples increased significantly with time (Figure 3), which obeyed a faster trend for the control sample, followed by Al‐, Al–CEO‐, Al–TiO_2_‐, and Al–TiO_2_–CEO‐treated meats, respectively. After 12 and 24 days of storage, the TVBN values of control were 26.58 and 43.19 mg N/100 g meat, respectively. However, these values were 15.21 and 25.31 (for Al), 12.12 and 23.41 (for Al–CEO sample), 11.47 and 19.24 (for Al–TiO_2_), and 8.68 and 13.27 (for Al–TiO_2_–CEO) N/100 g meat after 12 and 24 days of storage, respectively (*p* < .05). The upper acceptable limit for TVBN is 25 mg N/100 g (Alizadeh‐Sani et al., 2020), and the TVBN content of the control sample was more than this value on day 12 (26.58 mg N/100 g). However, the Al sample reached 25.31 on the 24th day, and the other treated samples had considerably lower values for TVBN than 25 mg N/100 g even on the 24th day, especially the Al–TiO_2_–CEO samples which had the smallest value (about 13 mg N/100 g).

Total volatile base nitrogen value in the meat samples is related to the decomposition of protein compounds to nonprotein nitrogen compounds as a result of bacteria activity and the proteolytic enzymes (Alizadeh‐Sani et al., 2020). In line with the microbial results, the antibacterial effect of CEO and TiO_2_ resulted in a lower activity of bacteria enzymes, and thereby, lower TVBN content of these samples. Similar results were obtained by Alizadeh‐Sani et al. (2020) on the addition of rosemary EO and TiO_2_ in refrigerated meat, Sayadi, Mojaddar Langroodi, Jafarpour (2021) on turkey meat coated with chitosan containing *Berberis vulgaris* extract and *Mentha pulegium* EO under MAP condition, Sayadi, Mojaddar Langroodi, Pourmohammadi et al. (2021) on using zein coating impregnated with ginger extract and *Pimpinella anisum* EO to prolong the shelf life of bovine meat, and Heydari‐Majd et al. (2019) on the application of poly lactic acid (PLA)/ZnO nanoparticles/*Zataria multiflora* Boiss. EO (ZEO) and *Menthe piperita* L. EO (MEO) on *Otolithes ruber* fillets.

### 3.5 Peroxide value

**FIGURE 4:**
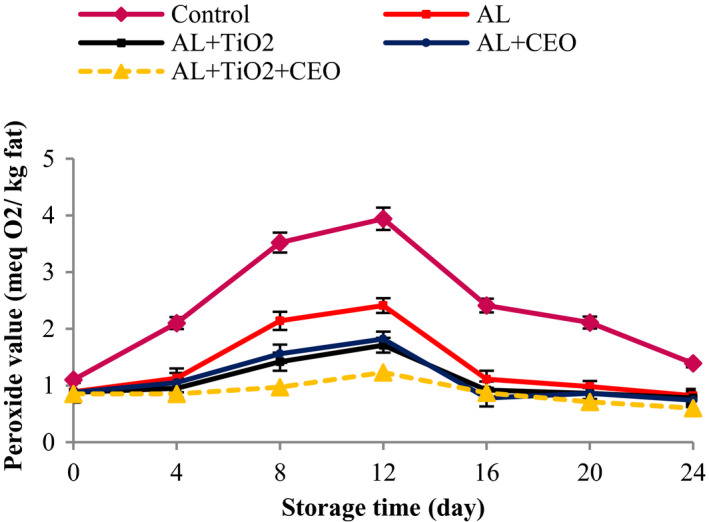
Changes in peroxide values of beef packed in alginate‐based films containing TiO_2_ and CEO at 4°C during 24 days of storage

The PV is usually measured to evaluate the chemical health status of product fat. After the formation of peroxides, the formation of volatile compounds (ketones, aldehydes, and alcohols) is accelerated, resulting in rancidity of fat and production of off‐flavor (Khezrian & Shahbazi, 2018). For this reason, PV measurement is considered a very important parameter. The initial PV of the samples was 1 meq peroxide/1000 g lipid, indicating the good quality of the initial beef. But the PVs increased steadily and reached the highest value until the 12th day (3.94 meq peroxide/1000 g lipid for the control), and then decreased until the end of the 24th day (Figure 4). An increase in the formation of PV caused an increasing trend in the PV curve up to day 12 and the decomposition of peroxide compounds from day 12 onward caused the curve to start a declining trend. This reduction was accompanied by a sudden increase in secondary products. The PV of the control was significantly higher than those of treated samples during the storage time. Up to the 12th day, the Al samples showed the highest PV after control, followed by Al–TiO_2_ or Al–CEO, and Al–TiO_2_–CEO‐treated meats, respectively. But from day 16 until the end of the storage time, there was no statistically significant difference among the treated samples. It is noteworthy that the changes in PV of Al–TiO_2_–CEO sample were very small and it increased from 1.00 to 1.23 meq peroxide/1000 g lipid (until the 12th day). However, this increase was from 1.00 to 1.76 meq peroxide/1000 g lipid for Al–TiO_2_ and Al–CEO samples.

According to the obtained results, the treatments of this study could effectively control the lipid oxidation of meat during 24 days of storage. The antioxidant activity of sodium alginate compound has been reported before (Sellimi et al., 2015). Sellimi et al. (2015) reported that sodium alginate extracted from Tunisian brown seaweed exerted 74% free radical scavenging activity at 0.5 mg/ml in DPPH radical scavenging activity test and demonstrated significant reducing activity (OD at 700 nm = 2) at 1.2 mg/ml in Ferric‐reducing activity test. These researchers showed that sodium alginate displayed a moderate ability to prevent bleaching of β‐carotene according to ß‐carotene–linoleic acid assay. In addition, sodium alginate, at 4 and 5 mg/ml, exerted potent scavenging activities (80% and 82%, respectively) according to hydroxyl radical scavenging activity test. Meanwhile, Al may act as an oxygen barrier (Nehchiri et al., 2021) between meat and its surroundings, resulting in the reduction in the lipid oxidation rate. On the other hand, it has been confirmed that CEO can reduce Fe^3^ ions effectively and scavenge the superoxide anion. This property comes from its polyphenolic compounds and good antioxidant activity of CEO (El‐Ghorab et al., 2010). The strong antioxidant activity of cuminal and γ‐terpinene has been reported. Meanwhile, monoterpene alcohols like linalool, terpineol, and pinocarveol have been indicated for CEO antioxidant activity (Abbdellaoui et al., 2019).

The antioxidant activity of TiO_2_ nanoparticles is confirmed by researchers (Alizadeh‐Sani et al., 2018). Alizadeh‐Sani et al. (2020) reported that TiO_2_ nanoparticles could turn the purple color of DPPH radical into yellow color of DPPH‐H, indicating that TiO_2_ has the ability to scavenge the free radical of DPPH, and therefore, can scavenge the produced free radicals during the oxidation reaction and therefore decrease the rate of oxidation. In our study, the synergistic effect of Al, CEO, and TiO_2_ in the prevention of lipid oxidation was evident. Similar results were also obtained on chilled meat coated with whey protein nanofibrils containing TiO_2_ nanotubes (Feng et al., 2019), fresh chicken packaged with gelatin film containing TiO_2_ nanoparticles and CEO (Sayadi, Amiri, et al., 2021), turkey breast meat coated with chitosan film containing 1% CEO (Taheri et al., 2018), and chicken breast fillet on application of silver nanoparticles in polyvinyl chloride films (Azlin‐Hasim et al., 2016).

### 3.6 Thiobarbituric acid

**FIGURE 5:**
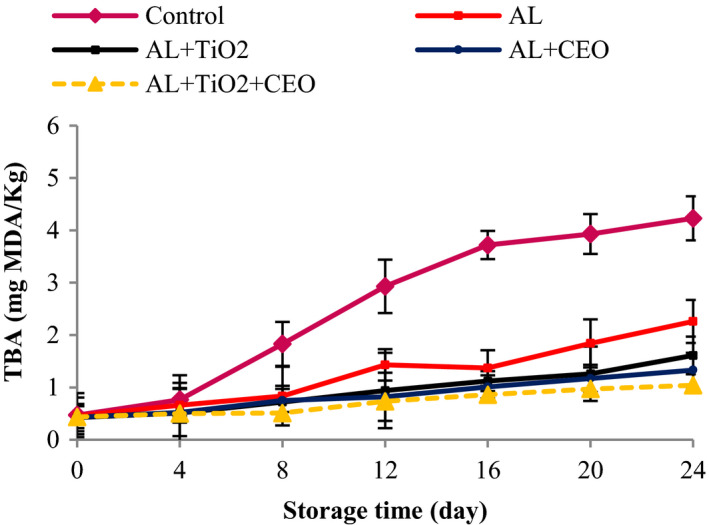
Changes in thiobarbituric acid values of beef packed in alginate‐based films containing TiO_2_ and CEO at 4°C during 24 days of storage

In the TBA test, MDA as an aldehyde compound is measured. Aldehydes are the secondary products of lipid oxidation, and their increase is an indication of lipid rancidity (Heydari‐Majd et al., 2019). The TBA of all samples increased significantly throughout 24 days of storage (Figure 5). The initial TBA value of beef was 0.45 mg MDA/kg, which was low and showed the freshness of the initial meat. The increasing trend in the TBA values of control was significantly much more than the treated samples, which reached 4.23 mg MDA/kg in the control samples after 24 days. However, the TBA values of Al, Al–TiO_2_, Al–CEO, and Al–TiO_2_–CEO reached 2.26, 1.61, 1.33, and 1.04 after 24 days (*p* < .05), respectively. The presence of strong antioxidant compounds in the CEO caused lower TBA values in Al–CEO sample than that of Al–TiO_2_. The physical barrier properties of Al to limit the oxygen penetration, the strong antioxidant properties of CEO due to its high phenolic content, and the free radical scavenging property of TiO_2_ are the reasons for the lower TBA values of the meat samples treated with Al, CEO, and TiO_2_ (Alizadeh‐Sani et al., 2020; Taheri et al., 2018). In line with PV results, from day 12 onward, with a decrease in the PVs, there was a large increase in the amount of TBA. Similar results were obtained by other researchers on turkey breast meat coated with chitosan containing 1% CEO (Taheri et al., 2018), *O. ruber* fillets packaged with nanocomposite film based on PLA/zinc oxide nanoparticle/ZEO and MEO (Heydari‐Majd et al., 2019), fresh pork and meat loins coated with alginate‐based film produced with turmeric (Bojorges et al., 2020), and beef wrapped with citric acid, cornstarch, and linear LDPE active films (Júnior et al., 2015).

### 3.7 Color analysis

Table 2 shows the color coordinates (L*, a*, and b*) of beef samples. According to Table 2, lightness (L*) decreased throughout the storage time which is related to the oxidation phenomenon that turns the color of meat from red to dark brown. Therefore, the increase in oxidation during the storage time resulted in a decrease in L* (lightness) and a* (redness) values of beef samples. The a* value is an indication of redness and is considered an important color parameter for meat and meat products. The increase in a* values is attributed to the oxygenation of the myoglobin which causes the red color of meat, and the reduction in a* values is the indication of myoglobin oxidation and formation of metmyoglobin, which turns the color of meat to brown (El Adab & Hassouna, 2016).

**TABLE 2:** Effect of alginate‐based films containing TiO_2_, CEO, and TiO_2_/CEO on color coordinates and sensory attributes of ground beef meat during 24 days of storage at refrigerated temperature

Sensory attributes	Treatment	Storage time (day)						
		0	4	8	12	16	20	24
L*	Control	36.20 ± 1.04^a^ *	35.20 ± 0.23^a^	32.11 ± 0.12^a^	30.18 ± 1.18^a^	29.82 ± 0.98^a^	27.71 ± 0.14^a^	25.15 ± 0.55^a^
	Al film	36.20 ± 1.04^a^	36.01 ± 0.42^b^	34.02 ± 0.35^b^	33.01 ± 0.15^b^	31.11 ± 0.74^b^	29.65 ± 0.31^b^	28.12 ± 0.35^b^
	Composite Al‐based films	36.20 ± 1.04^a^	36.00 ± 0.55^b^	35.02 ± 0.45^c^	34.01 ± 0.25^c^	33.11 ± 0.74^c^	32.45 ± 0.41^c^	31.87 ± 0.24^c^
a*	Control	23.87 ± 0.45^a^	20.21 ± 0.87^a^	16.10 ± 0.48^a^	14.21 ± 0.95^a^	12.21 ± 0.48^a^	8.45 ± 0.98^a^	6.12 ± 0.42^a^
	Al film	23.87 ± 0.45^a^	22.22 ± 0.42^b^	20.12 ± 0.31^b^	19.00 ± 0.12^b^	18.23 ± 0.34^b^	16.42 ± 0.44^b^	13.22 ± 0.55^b^
	Composite Al‐based films	23.87 ± 0.45^a^	22.10 ± 0.35^b^	21.11 ± 0.45^c^	20.32 ± 0.34^c^	19.10 ± 0.25^c^	17.52 ± 0.54^c^	15.22 ± 0.65^c^
b*	Control	12.76 ± 0.45^a^	10.93 ± 0.41^a^	10.01 ± 0.21^a^	9.22 ± 0.61^a^	9.00 ± 0.25^a^	8.86 ± 0.87^a^	8.12 ± 0.12^a^
	Al film	12.76 ± 0.45^a^	12.12 ± 0.24^b^	11.00 ± 0.33^b^	10.20 ± 0.42^b^	9.81 ± 0.46^b^	9.11 ± 0.42^b^	9.00 ± 0.35^b^
	Composite Al‐based films	12.76 ± 0.45^a^	12.00 ± 0.34^b^	11.50 ± 0.44^c^	11.20 ± 0.24^c^	10.82 ± 0.54^c^	10.11 ± 0.42^c^	9.54 ± 0.42^c^
	Control	9 ± 0^Aa^	7.6 ± 0.53^Bc^	5.2 ± 0.36^Cd^	3.1 ± 0.23^Db^	1.7 ± 0.51^Ec^	0 ± 0^Fd^	0±0^Fd^
	AL	8.9 ± 0.23^Aa^	8.6 ± 0.68^Aa^	8.1 ± 0.32^Ba^	4.2 ± 0.36^Ca^	3.2 ± 0.0^Cb^	1.1 ± 0.42^Dc^	0.3 ± 0.62^Ec^
Color**	AL + TiO_2_	8.8 ± 0.33^Aa^	8.8 ± 0.73^Aa^	8.2 ± 0.34^Ba^	4.3 ± 0.51^Ca^	3.6 ± 0.33^Da^	2.3 ± 0.21^Eb^	1.6 ± 0.28^Fb^
	AL + CEO	8.9 ± 0.72^Aa^	8.6 ± 0.56^Ba^	8.0 ± 0.62^Ca^	4.1 ± 0.39^Da^	3.5 ± 0^Ea^	2 ± 0^Fb^	1.3 ± 0.62^Gb^
	AL + TiO_2_ + CEO	8.9 ± 0.22^Aa^	8.8 ± 0.29^Ba^	8.2 ± 0.36^Ca^	4.3 ± 0.44^Da^	3.6 ± 0.63^DEa^	2.9 ± 0.37^Ea^	2.3 ± 0.48^Ea^
	Control	9 ± 0^Aa^	7.7 ± 0.52^Bc^	6.3 ± 0.54^Cc^	4.0 ± 0^Dd^	1.4 ± 0.56^Ec^	0 ± 0^Fd^	0 ± 0^Fd^
	AL	8.9 ± 0.23^Aa^	8.5 ± 05^Aa^	7.2 ± 0.51^Bb^	6.2 ± 0.52^Ccb^	5.3 ± 0.65^Dab^	4.1 ± 0.43^Eb^	2.6 ± 0.31^Fc^
Odor	AL + TiO_2_	9 ± 0^Aa^	8.7 ± 0.23^Aa^	7.5 ± 0^Ba^	6.4 ± 0.62^Ca^	5.7 ± 0.28^Da^	4.5 ± 0.36^Ea^	3.8 ± 0.11^Fb^
	AL + CEO	8.5 ± 0^Ab^	8.2 ± 0.68^Ab^	7 ± 0^Bb^	5.9 ± 0.51^Cc^	4.9 ± 0.22^Db^	3.7 ± 0.53^Ec^	2.8 ± 0.24^Fc^
	AL + TiO_2_ + CEO	8.5 ± 0.56^Aa^	8.1 ± 0.58^Ab^	7.2 ± 0^Bb^	6 ± 0^Cbc^	5.1 ± 0.82^Dab^	4.2 ± 0.51^Eb^	4 ± 0^Ea^
	Control	9 ± 0^Aa^	6.2 ± 0.56^Bc^	3.6 ± 0.37^Cc^	1.7 ± 0.43^Dc^	1.2 ± 0.46^ECc^	0 ± 0^Fc^	0 ± 0^Fd^
	AL	8.9 ± 0.25^Aa^	8.4 ± 0.41^Ba^	7.5 ± 0^Cb^	5.9 ± 0.46^Db^	5.4 ± 0.32^Eb^	3.6 ± 0.25^Fb^	3.5 ± 0.24^Fc^
Overall acceptability	AL + TiO_2_	9 ± 0^Aa^	8.7 ± 0.64^Aa^	7.9 ± 0.36^Ba^	6.5 ± 0.73^Ca^	6 ± 0^Ca^	3.9 ± 0.42^Db^	3.7 ± 0.27^Dc^
	AL + CEO	8.8 ± 0.42^Aa^	8.1 ± 0.62^Bb^	8 ± 0^Ba^	6.6 ± 0.26^Ca^	6 ± 0^Da^	3.8 ± 0.53^Eb^	3.6 ± 0.51^Ec^
	AL + TiO_2_ + CEO	8.9 ± 0.42^Aa^	8.2 ± 0.32^Bb^	8 ± 0^Ba^	6.7 ± 0.72^Ca^	6.1 ± 0.43^Ca^	5.4 ± 0^Da^	5 ± 0^Db^

* Data with different small letters in each column are significantly different (*p* < .05) for color coordinates.

** Data of sensory evaluation with different capital and small letters in each row and column are significantly different (*p* < .05).

The L* and a* values reduced from 36.20 to 25.15 and 23.87 to 6.12 at the end of the storage time for the control, respectively, while this reduction was to 31.87 for L* and 15.22 for a* value in the treated samples. This indicates the effect of active packaging and nanocomposite films containing antimicrobial and antioxidant compounds in retarding the formation of the undesirable metmyoglobin pigment and maintaining the color quality of meat for a longer time. The results of L* and a* values, in this study, were in line with the findings of Takma and Kore (2019) on the addition of chitosan and alginate layer to chicken breast meat, and Sirocchi et al. (2017) on the application of rosemary EO and modified atmosphere packaging to beef.

In relation to b* values, the indicator of “yellowness,” a significant decrease occurred for all the samples. The reduction in b* is attributed to a decrease in the oxymyoglobin content and an increase in the formation of metmyoglobin in the meat. The reduction in the oxymyoglobin content occurred due to the consumption of oxygen by microorganisms (El Adab & Hassouna, 2016). In this regard, the treated samples with nanocomposite films showed lower b* values than that of the control as a result of the antioxidant compounds of CEO and radical scavenging properties of TiO_2_ that retarded the oxidation phenomenon (El Adab & Hassouna, 2016). No significant difference was observed among the color coordinates of the Al + CEO, Al + TiO_2_, and Al + TiO_2_ + CEO samples; meanwhile, the color coordinates of Al samples were placed after the control. According to the results, the incorporation of TiO_2_ and CEO into the alginate‐based films could significantly enhance the stability of color in the meat samples. The results of color parameters variations in this study were in good agreement with the results of El Adab and Hassouna (2016) on the addition of oregano and thyme EOs in fermented poultry meat sausage, Heydari et al. (2020) on the application of Qodume Shirazi seed mucilage‐based edible coating containing lavender essential oil on the fresh ostrich meat, and Tosati et al. (2017) on the use of bovine gelatin and turmeric starch on frankfurter sausage.

### 3.8 Sensory properties

All sensory attributes of the meat samples (color, odor, and overall acceptability) declined throughout the storage time (Table 2). This reduction trend was much faster in the control sample than in the treated samples during the storage time, and hence, the obtained score for color, odor, and overall acceptance was zero for the control sample on day 20.

Regarding the color parameter, at the beginning of storage, there was no significant difference among the treated samples. However, by moving toward the end of the storage time, the Al sample obtained lower scores than the other treated samples and the Al + TiO_2_ + CEO sample received the highest score. Regarding the odor parameter, it seems that the treated samples without CEO obtained a higher score than the samples containing CEO. This can be due to the effect of the CEO smell. It is noteworthy that although the odor parameter score was significantly lower in the CEO‐containing samples over time, this difference was small. Another important point was that by moving toward the end of the storage time, this difference disappeared, which could be due to the decrease in CEO odor over time, and finally, the Al + TiO_2_ + CEO samples obtained the highest score. In terms of overall acceptance, it was observed that the score obtained by the treated samples throughout the storage time was significantly higher than that obtained by the control sample. Meanwhile, the scores obtained at the beginning of storage were not much different for all treatments, but toward the end of the storage time, the Al + TiO_2_ + CEO sample obtained the highest scores. The antimicrobial effect of Al, CEO, TiO_2_, and Al + TiO_2_ + CEO induced the enhancement of sensory attributes in the treated meats compared to the control. Our results are consistent with those of Alizadeh‐Sani et al. (2017) and Azizi‐Lalabadi et al. (2020). In these studies, it was demonstrated that the application of TiO_2_ nanoparticles effectively improved the sensory attributes of white shrimp and lamb, respectively. Sharafati Chaleshtori et al. (2016) and Taheri et al. (2018) declared that the application of chitosan containing CEO resulted in good overall acceptance in chicken meat and turkey breast meat, respectively. Similar results were obtained by other researchers (Chen et al., 2016; Diao et al., 2020; Sayadi, Amiri, et al., 2021).

### 3.9 The estimation of shelf life for beef samples

The shelf life of beef samples was estimated according to the results of microbial and chemical tests (Table 3). In terms of TVC, TBA, PV, a*, and TVBN results, all treated samples were acceptable until day 24, while this number was 8 for the control. However, the pH and sensory evaluation tests limited the shelf life of control to 4 days. Although the treated samples were sensually acceptable up to day 16, the pH test reduced the shelf life of the Al sample to 8 days and Al + CEO and Al + TiO_2_ samples to 12 days. However, the pH of Al + TiO_2_ + CEO sample did not exceed the upper limit until the 24th day. Overall, the shelf life of control, Al, and composite films was around 4, 8, and 16 days, respectively.

**TABLE 3:** The estimation of beef shelf life according to data obtained from physicochemical, microbial, and sensorial experiments during 24 days of storage

	Shelf life (day)		
	Control	Alginate film	Composite alginate‐based films
Total viable count^a^	8	24	Not achieved
Peroxide value	Not achieved (3.94 at day 12)	Not achieved (2.83 at day 12)	Not achieved [1.86 (in average) at day 12]
TBA value	~8	~20	Not achieved
TVBN value	8	20	Not achieved
a*^c^	8	24	24
Sensorial^c^	4	16	16
pH	4	8	12 (and until 24 days for Al + TiO_2_ + CEO sample)

^a^ According to Iran National Standards Organization, the upper limit value for TVC in fresh beef is 5 × 10^5^ log CFU/g, 5 meq/kg fat is the upper limit value for peroxide value, 25 mg N/100 g is the upper limit value for TVBN, 5.8 is the upper limit value for pH, and no upper limit value is defined for TBA, but according to literature [Alizadeh Sani et al. (2020)], 2 mg MDA/1000 g is the upper limit value for TBA.

^b^ According to the color analysis results.

^c^ According to the sensory evaluation results and overall acceptance score more than 4.

## 4 CONCLUSION

In this study, nanocomposite alginate‐based films containing TiO_2_ nanoparticles and CEO (as antioxidant and antimicrobial agents), alone or in combination, were fabricated. The produced active packaging films could remarkably reduce the lipid oxidation and microbial spoilage, improve the color quality and sensory attributes, and increase the shelf life of fresh beef. In this regard, the combination use of TiO_2_ and CEO resulted in better results compared to the sole use of TiO_2_ and CEO. The results of this study showed that the use of Al + TiO_2_ + CEO as a novel nanocomposite film is greatly beneficial in preserving the quality parameters of fresh beef.

## CONFLICT OF INTEREST

Mehran Sayadi, Ali Mojaddar Langroodi, Sedigheh Amiri, and Mohsen Radi declare that they have no conflict of interest.

## AUTHOR CONTRIBUTIONS

**Mehran Sayadi:** Funding acquisition (lead); Investigation (equal); Methodology (equal). **Ali Mojaddar Langroodi:** Conceptualization (equal); Investigation (equal); Methodology (equal); Visualization (equal). **Sedigheh Amiri:** Investigation (supporting); Methodology (equal); Visualization (equal); Writing – original draft (lead); Writing – review & editing (equal). **Mohsen Radi:** Methodology (equal); Supervision (equal); Writing – review & editing (equal).

## ETHICAL APPROVAL

This article does not contain any studies with human participants or animals performed by any of the authors.

## Data Availability

Data will be made available upon reasonable request.
